# Bacitracin Methylene Disalicylate (BMD) Treatment Affects Spleen Proteome in Broiler Chicks Infected with *Salmonella enteritidis*

**DOI:** 10.3390/antibiotics13050414

**Published:** 2024-05-01

**Authors:** Adedeji Adetunji, Theresa Casey, Uma K. Aryal, Tunde Ogundare, Jackeline Franco, Yewande Fasina

**Affiliations:** 1 Department of Animal Sciences, North Carolina Agricultural and Technical State University, Greensboro, NC 27411, USA; adetunjia@uapb.edu (A.A.);; 2 Department of Agriculture, University of Arkansas at Pine Bluff, Pine Bluff, AR 71601, USA; 3 Department of Animal Sciences, Purdue University, West Lafayette, IN 47907, USA; 4 Purdue Proteomics Facility, Bindley Bioscience Center, Purdue University, West Lafayette, IN 47907, USA; 5 Department of Comparative Pathobiology, Purdue University, West Lafayette, IN 47907, USA

**Keywords:** bacitracin methylene disalicylate, proteomics, broiler chicks, spleen, *Salmonella enteritidis*

## Abstract

Bacitracin Methylene Disalicylate (BMD), as a feed additive to poultry diets, enhances digestion, prevents *Salmonella enteritidis* (SE) colonization, and treats current infections. The objective of this study was to utilize a quantitative proteomic approach to determine the effect of BMD feed additive on broiler chickens challenged with SE in the spleen proteome. At 1 d of age, chicks were randomly allocated into four groups: control with and without SE challenge (CON, n = 60; CON-SE, n = 60), BMD with and without SE challenge (BMD, n = 60; BMD-SE, n = 60). Birds in the CON-SE and BMD-SE treatment were administered SE inoculum by oral gavage. On day three and day seven post-gavage, the spleen was collected aseptically from birds in each treatment group (CON, n = 4/day; CON-SE, n = 4/day; BMD, n = 4/day; BMD-SE, n = 4/day). Proteomic analysis by liquid chromatography-tandem mass spectrometry (LC-MS/MS) showed an increased abundance of 115 proteins and decreased of 77 due to the BMD. Proteins that decreased in abundance were enriched for fibrinogen complex and extracellular space, whereas proteins that increased in abundance were enriched for proteasome-mediated ubiquitin-dependent protein catabolic process and mitochondrion. Analysis of the interaction between BMD and the *Salmonella* challenge found 230 differentially abundant proteins including proteins associated with RNA binding, spliceosome, protein transport, and cell adhesion among the upregulated proteins, and those associated with protein folding, carbon metabolism, biosynthesis of nucleotide sugars, response to oxidative stress, positive regulation of NIK/NF-kappaB signaling, and inflammatory response among the downregulated proteins. The impact of BMD treatment on spleen proteome indicates an anti-apoptotic effect. BMD also modified the response of the spleen to the SE challenge with a marked decrease in proteins that prompt cytokine synthesis and an increase in proteins involved in the selective removal of unfolded proteins.

## 1. Introduction

*Salmonella enteritidis* (SE) infections cause great economic loss to poultry producers, as well as food borne gastroenteritis in humans in developed and developing countries [1,2]. This is because the bacterium can easily be transmitted via contaminated meat and egg products [3]. Although SE infections are primarily contained within the gastrointestinal tract, during infection there is an increase in the cellularity of the spleen. The spleen is part of the immune system and functions to remove bacteria from the bloodstream through the activity of leukocytes housed there. During an active infection spleen cellularity increases through the proliferation and recruitment of phagocytes and lymphocytes to prevent systemic colonization of the disease [4,5]. Our previous analysis of the effect of an SE challenge on the spleen proteome indicated that infection in growing broiler chicks is metabolically costly. Protein signatures indicated that during an SE challenge, energy is diverted from normal developmental processes to potentiate disease resistance mechanisms [6].

Over the previous decades, poultry producers have relied on feeding antibiotics prophylactically to mitigate the negative effects of *Salmonella* infections on broiler health and development. A common antibiotic used as a growth promoter in poultry is Bacitracin Methylene Disalicylate (BMD). As a feed additive, BMD enhances digestion in the small intestine of growing broilers and prevents SE colonization, as well as treats current infections. Mechanistically, BMD acts by interfering with protein synthesis and bacterial cell wall synthesis, resulting in microbial cell rupture and death [7,8].

While many studies have consistently shown that in-feed bacitracin is beneficial to the health of broiler chickens [9,10], there is a global call to cease the use of in-feed antibiotics [11]. This is a result of concerns about food safety, antibiotic residue, and the spread of drug-resistant bacterial pathogens [12,13,14]. Understanding the mechanism by which bacitracin helps in the prevention and treatment of growing animals challenged with SE can help in the development of alternative approaches to prevent diseases and promote the overall growth and health of poultry [1,15,16]. To this end, we utilized liquid chromatography–tandem mass spectrometry (LC-MS/MS) shotgun proteomics to determine the effect of BMD feed additive on broiler chickens challenged with SE in the proteome of the spleen. The proteomic profiles of BMD will help provide fundamental data for determining the biochemical characteristics of potential alternatives to antibiotics and deciphering genes that can serve as potential therapeutic targets for the control of bacteria.

## 2. Results

### 2.1. Performance Parameters and Concentration of SE in the Ceca of Broiler-Chickens

The concentration of SE in the ceca was analyzed using a completely randomized design having four treatment groups. Data analysis was conducted using a one-way ANOVA, and significant differences among means were identified using the Tukey option of the general linear model (GLM) procedure as a post hoc test. Results are presented as means with standard error of the mean (SEM) and statements of statistical significance were determined at a significance level of *p* < 0.05. The chicks in the CON-SE- and BMD-SE-challenged groups had 5.68 and 3.39 log_10_ CFU SE/g cecal content on d 3, and 4.17 and 2.41 log_10_ CFU SE/g cecal content on d 7, respectively, whereas SE was undetected in chicks in the CON and BMD treatments (Table 1) [1].

### 2.2. Overall Effect of BMD Treatment on Protein Abundance

**Figure 1:**
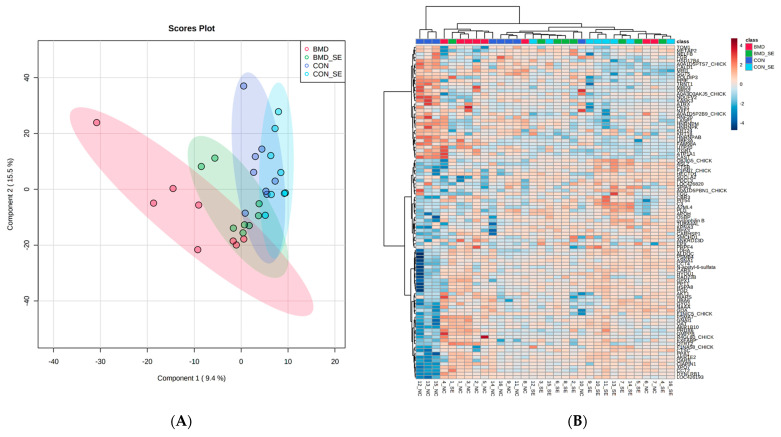
PLSDA (**A**) and the heat map with dendrogram resulting from hierarchical cluster analysis (**B**) of proteins extracted from spleen samples obtained from BMD, BMD-SE, CON, and CON-SE group broiler chickens (n = 4). The PLSDA score plot demonstrates distinct sample clusters based on BMD, BMD-SE, CON, and CON-SE groups, while the heat maps visualize the 100 most distinguishing proteins, with greater abundance indicated in red and lesser abundance in blue, relative to BMD (red), BMD-SE (green), CON (blue), and CON-SE (light blue) groups.

Principal component and hierarchical cluster analyses indicated that BMD treatment (BMD-CON) and BMD treatment in the presence of SE infection (BMDSE-CONSE interaction) affected protein expression profiles (Figure 1). However, the day post-gavage SE did not affect spleen proteome and so it was removed from the model (see Supplementary Material).

The BMD treatment had an overall effect on the abundance of 77 proteins at a false discovery rate of less than 5% (FDR < 0.05). Relaxing the stringency to FDR < 0.1 identified 192 proteins affected by the BMD treatment (Table 2). To cast a wide enough net for meaningful functional annotation analysis, the set of proteins that passed the FDR < 0.1 stringency was used. Of these 192 proteins, 115 proteins increased and 77 decreased in abundance in response to BMD treatment. Proteins that decreased in abundance enriched the category fibrinogen complex, extracellular space, and signal. Proteins that increased in abundance were categorized as proteasome-mediated ubiquitin-dependent protein catabolic process, nucleotide excision repair, ATP binding, and mitochondrion (Table 3).

We investigated molecular and biological interactions in the proteins affected by BMD treatment using IPA-based protein network analysis. The top-enriched network based on the high percentage of focus molecules in our data set is shown in Table 4. Network 1, with the highest score (53), indicated that BMD had anti-apoptotic effects and suppressed immune gene response by increasing abundance of cytokine-induced apoptosis inhibitor 1 (CAIPIN1) and scaffold attachment factor B2 (SAFB2). Linker genes such as T1A1 involved in inducing apoptosis and EWSR1 were lower in abundance. The downregulation of EWSR1 was found to be linked with the upregulation of HECTD1, which is involved in the proteasomal ubiquitin-dependent protein catabolic process. Network 4 had 35 focus proteins containing the 19S, 20S, and 26S proteasome family indicating that BMD adopts the proteasomal ubiquitin-dependent protein catabolic process in the maintenance of protein homeostasis by selectively removing misfolded or damaged proteins that could affect cell/tissue/organ function. Interestingly, with the direct activation of proteasome proteins and HPT1 involved in negatively regulating CD4 T lymphocytes, Hsp70 was indirectly inhibited. This also indicates that there is a relationship between the proteasomal ubiquitin-dependent protein catabolic process and the inflammatory process.

**Table 2:** Summary of number of genes differentially expressed at *p*-value cut-off and associated maximal FDR.

Three-Way ANOVA	FDR < 0.05	FDR < 0.1
BMD	77	192
Treatment X SE Challenge	126	230

**Table 3:** Representative gene sets within the functional groups significantly enriched (*p* < 0.05) with genes expressed by overall BMD, and interaction between treatment and challenge identified using Functional Annotation Clustering in NIH DAVID.

Term	%	*p*-Value	Genes
**Proteins that decreased with the BMD**			
Fibrinogen Complex	5.2	8.0 × 10^−07^	FN1, FGA, FGG, FGB,
Extracellular Space	14.3	9.1 × 10^−03^	ORM1, LOC426820, DCN, FGB, FN1, C3, COL12A1, FGA, COL6A1, SPIA3, FGG
Signal	24.7	4.2 × 10^−02^	DCN, FKBP9, FGB, FN1, COL12A1, COL6A1, VNN1, ARSB, ORM1, CD44, C3, FGA, SPIA3, VTN, ITIH3, FGG, ELN, MIA3, TM9SF2
**Proteins that increased with the BMD**			
Proteasome-mediated ubiquitin-dependent protein catabolic process	6.9	4.7 × 10^−05^	PSMA7, RAD23B, PLAA, PSMB4, PSMC5, PSMD12, PSMB2, RAD23A
Nucleotide excision repair	3.4	1.2 × 10^−02^	RFC2, RAD23B, RAD23A, RPA3
ATP binding	14.7	2.8 × 10^−02^	SMCHD1, SKIV2L2, PSMC3, NME3, MSH2, SYK, CTPS1, CCT2, PSMC5, VRK1, ASNA1, RFC2, UBA6, R4GL80_CHICK, HYOU1, AKT1
Mitochondrion	5.2	5.7 × 10^−02^	BID, CIAPIN1, CS, QTRT2, NDUFB4, SAMM50
**Proteins decreased with the treatment X challenge**			
Protein folding	6.2	1.2 × 10^−05^	CCT2, PPIA, CALR, CALR3, HSP90B1, PPIB, HYOU1, P4HB
Carbon metabolism	5.4	2.4 × 10^−03^	GPI, TKTL1, CS, IDH2, ADH4, MDH2, FH
Biosynthesis of nucleotide sugars	3.9	1.6 × 10^−03^	GPI, TSTA3, GMPPB, GFPT1, GMPPA
Response to oxidative stress	3.1	1.3 × 10^−02^	PRDX6, GPX1, ATOX1, PRDX4
Positive regulation of NIK/NF-kappaB signaling	2.3	4.0 × 10^−02^	CALR, CALR3, RELA
Inflammatory response	4.7	6.7 × 10^−03^	PLAA, MAP2K3, GGT5, RELA, AKT1, EXFABP
**Term**	**%**	***p*-Value**	**Genes**
**Proteins that increased with the treatment X challenge**			
RNA binding	12.7	1.7 × 10^−04^	RBM23, RPS27, RPL3, FUBP3, SRSF6, POLDIP3, HNRNPU, CPSF6, HNRNPR, MATR3, SRSF7, NONO, CRNKL1
Spliceosome	7.8	1.3 × 10^−04^	HNRNPU, CDC5L, SRSF6, PPIE, HSPA8, SRSF7, CRNKL1, EFTUD2
Peptidyl-prolyl cis-trans isomerase activity	3.9	2.8 × 10^−03^	PIN4, PPIE, FKBP15, FKBP3
Protein transport	7.8	1.6 × 10^−03^	VPS45, SEC16A, TOM1, NUP93, AP2M1, VPS16, NUP98, F1NLW3_CHICK
Cell adhesion	5.9	3.0 × 10^−02^	LAMA5, CTNNA2, ARVCF, AFDN, LAMC1, NID1

**Table 4:** Biological functions of genes in the top five networks generated in IPA; (scores) and [central molecules] within networks.

**Figure 2:**
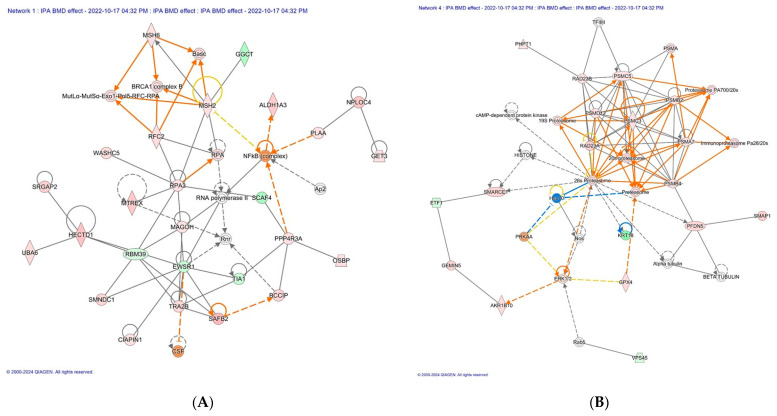
The top scoring IPA protein networks DNA replication, recombination, and repair, cancer, gastrointestinal disease (**A**), infectious diseases, organismal injury and abnormalities, and cancer (**B**) are depicted for overall BMD treatment. In the figure, red denotes upregulation while green indicates downregulation, with color intensity reflecting the relative magnitude of change in protein expression. Solid lines represent direct interactions, whereas dashed lines signify indirect interactions. The protein interaction networks were constructed using QIAGEN Ingenuity Pathway Analysis, https://www.qiagenbioinformatics.com/products/ingenuity-pathway-analysis/ (accessed on 17 October 2022).

**Figure 3:**
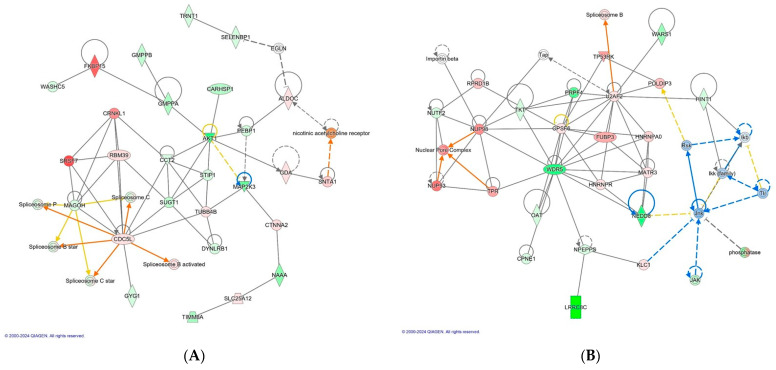
The top scoring IPA protein networks cancer, dermatological diseases and conditions, RNA post-transcriptional modification (**A**), cellular assembly and organization, molecular transport, and RNA trafficking (**B**) are depicted for BMD treatment X *Salmonella* challenge. In the figure, red denotes upregulation while green indicates downregulation, with color intensity reflecting the relative magnitude of change in protein expression. Solid lines represent direct interactions, whereas dashed lines signify indirect interactions. The protein interaction networks were constructed using QIAGEN Ingenuity Pathway Analysis, https://www.qiagenbioinformatics.com/products/ingenuity-pathway-analysis/ accessed on 17 October 2022.

**Overall BMD Effect**
DNA Replication, Recombination, and Repair, Cancer, Gastrointestinal Disease (CIAPIN1, 53) (Figure 2A)
Cellular Assembly and Organization, Hereditary Disorder, Organismal Injury and Abnormalities (AKT1, and RPL34, 53)
Cell Death and Survival, Cell-To-Cell Signaling and Interaction, Cellular Assembly and Organization (CCT2 and FN1, 35)
Infectious Diseases, Organismal Injury and Abnormalities, Cancer (19S, 20S, and 26S proteasome family, 32) (Figure 2B)
Behavior, Cell Signaling, Cancer (ELN, PRDX6, 32)
**Treatment X Challenge**
Cancer, Dermatological Diseases and Conditions, RNA Post-Transcriptional Modification (CARHSP1, 56) (Figure 3A)
Cellular Function and Maintenance, Lipid Metabolism, Small Molecule Biochemistry (HYOU1 and LDHA, 53)
Cardiovascular System Development and Function, Organismal Injury and Abnormalities, RNA Post-Transcriptional Modification (EFTUD2, 50)
Cellular Assembly and Organization, Molecular Transport, RNA Trafficking (HINT1, 45) (Figure 3B)
Cell Cycle, Gene expression, Infectious Diseases (FKBP3 and AIMP1, 35)

### 2.3. Effect of Interaction between BMD Treatment and Salmonella Challenge on Protein Abundance

Analysis of the interaction between BMD and the *Salmonella* challenge found an abundance of 126 proteins were altered at FDR < 0.05 and 230 proteins at FDR < 0.1. As above, the set of proteins that met the criteria of being altered by the interaction of BMD and SE at FDR < 0.1 was used for down-stream analysis. Proteins increased in abundance enriched RNA binding, spliceosome, peptidyl-prolyl cis-trans isomerase activity, protein transport and cell adhesion. Proteins that decreased in abundance enriched the categories protein folding, carbon metabolism, biosynthesis of nucleotide sugars, response to oxidative stress, positive regulation of NIK/NF-kappaB signaling, and inflammatory response (Table 3).

Moreover, IPA analysis also revealed that BMD treatment and the SE challenge interaction group induced the upregulation of proteins such as CDC5L, CRNKL1, SRSF7 and RBM39 involved in pre-mRNA splicing. This is of interest because in our previous study, the *Salmonella* challenge did not induce spliceosome activity, it was rather observed in the control group without a *Salmonella* infection. Therefore, this can be attributed to BMD action. However, it is notable that the linkage proteins associated with heat shock proteins such as SUGT1 and MAGOH were downregulated. Network 4 (score, 45) revealed that BMD treatment X SE challenge downregulated LRRC8C and HINT1, a key component of the interleukin-6 (IL-6)/JAK1/STAT3 immune and inflammation response. Intriguingly, these linkage proteins of HNT1 and LRRC8C (JNK, RSK, and IKK family) were also predicted to be inhibited by IPA. The downregulation of HINT1 and upregulation of U2AF2 also leads to the activation of spliceosome B.

### 2.4. IPA Canonical Pathways Enriched with Differentially Abundant Proteins

**Figure 4:**
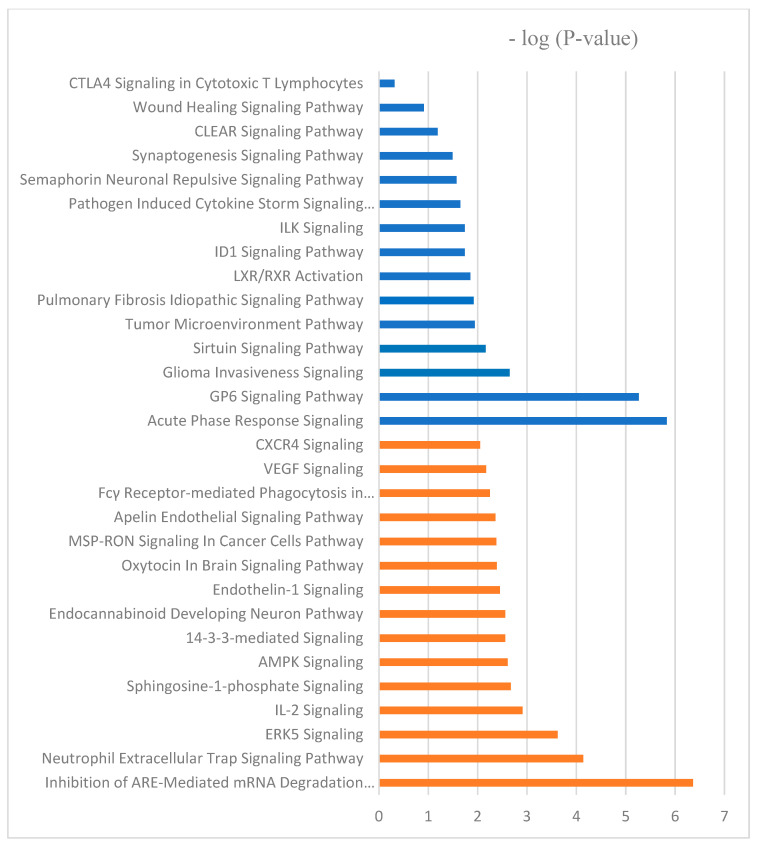
The molecular and cellular function ontologies of proteins affected by BMD–SE treatment in the spleen established through IPA analysis. The *Y*-axis represents the negative logarithm of the *p*-value. The functional analyses were generated through the use of QIAGEN Ingenuity Pathway Analysis, https://www.qiagenbioinformatics.com/products/ingenuity-pathway-analysis/ accessed on 17 October 2022.

Canonical pathways can be described as well-defined biochemical processes in the cell that result in a specific functional biological consequence. The top five canonical pathways enriched with proteins differentially abundant in spleen of CON versus BMD treated chicks were the FAT10 signaling pathway, the inhibition of ARE-mediated mRNA degradation pathway, acute phase response signaling, the role of tissue factor in cancer, and the GP6 signaling pathway (Table 5). IPA software (https://digitalinsights.qiagen.com/?cmpid=CM_QDI-GA-Sitelink&gad_source=1&gclid=EAIaIQobChMI4v-G963rhQMVlNcWBR0Gzg80EAAYASABEgI9X_D_BwE, accessed on 1 April 2024) predicted the activation of the pathways: inhibition of ARE-mediated mRNA degradation pathway and ERK signaling. Acute phase response signaling, GP6 signaling pathway, and pathogen-induced cytokine storm signaling pathway were predicted to be inhibited canonical pathways (Figure 4).

Furthermore, the top five canonical pathways according to the number of differentially abundant proteins in the BMD treatment and SE challenge interaction group include spliceosomal cycle, colanic acid building blocks biosynthesis, GDP-mannose biosynthesis, TCA cycle II (eukaryotic), glutathione redox reactions I. spliceosomal cycle, and PPARα/RXRα activation were activated by BMD treatment X SE challenge; however, acute phase response signaling, and IL-17 signaling were inhibited (Figure 4).

### 2.5. Activated and Inhibited Proteins Identified by Upstream Regulator Analysis

IPA upstream functional analysis was used to predict the top upstream regulatory molecules that inhibited or activated downstream proteins based on changes in response to BMD treatment and interaction between BMD treatment and the SE challenge. The activation z-score algorithm was used to make predictions. The IPA predicted the following upstream molecules were activated in our overall BMD data set: prostaglandin J2 (*z*-score = 2), CLDN7 (*z*-score = 2.22), EGFR (*z*-score = 2.43), ERBB2 (*z*-score = 2.18), and ADCYAP1 (*z*-score = 2). The following were predicted to be inhibited upstream regulators by IPA algorithms: IL6 (*z*-score = −2.69), IL4 (*z*-score = −2.36), AGT (*z*-score = −2.24), and CCR2 (*z*-score = −2.24). In the analysis of BMD in the presence of *Salmonella* infection (treatment x challenge), IPA predicted the following upstream regulators were inhibited: NFE2L2 (*z*-score = −2.15), XBP1 (*z*-score = −2.91), CD28 (*z*-score = −2.14), CD40 (*z*-score = −2.53), CD3 (*z*-score = −2.24), and lipopolysaccharide (*z*-score = −1.73), whereas, dexamethasone (*z*-score = 2.03), (CEBPA (*z*-score = 2.41), and ESRRG (*z*-score = 2.19) were activated. Interestingly, network analysis by IPA identified cytokines (IL6 and IL4) and lipopolysaccharide as potential upstream regulators that were inhibited in the BMD group and BMD treatment X *Salmonella* challenge group, respectively, suggesting that BMD, with or without the presence of infection, prevented inflammation and accompanying cytokine recruitment.

## 3. Discussion

The inclusion of BMD in feed modifies innate immune responses and improves the growth performance of broiler chickens [1,17]. This improvement is associated with BMD-induced changes to the bacterial microbiome of the ceca, where it promotes the growth of beneficial bacteria and resultantly improves intestinal function [18]. The spleen is an immune organ, and it filters microorganisms from the blood through the activity of leukocytes housed there. The overall effect of BMD treatment on spleen proteome reflected a reduction in the inflammatory response and enhanced cell redox homeostasis. The top five IPA networks for BMD treatment consists of proteins that confer anti-apoptotic effects on the cell, regulate gene expression, and mitigate cytokine recruitment. BMD treatment also modified the response of the spleen to the SE Challenge. In a previous analysis, we found the *Salmonella* challenge modified the spleen proteome by increasing the abundance of PRDX6 and CTSS proteins, which are involved in response to cellular stress and lysosomal proteins, respectively. Analysis of the effect of BMD on the spleen proteome revealed a marked decrease in proteins that prompt cytokine synthesis and an increase in proteins involved in the selective removal of unfolded proteins via the proteasome-mediated ubiquitin-dependent protein catabolic process.

**Figure 5:**
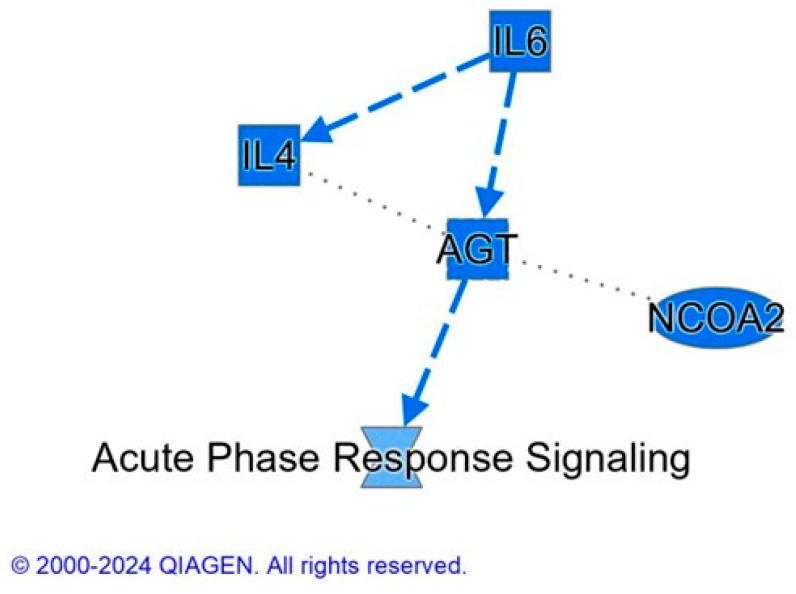
IPA summary graph of the effect of BMD on spleen proteome. Blue color indicates a prediction that the following molecules: IL6 (interleukin-6), IL4 (interleukin-4), AGT (angiotensinogen), NCOA2 (nuclear receptor co-activator 2), and acute phase response signaling are inhibited. The protein interaction networks were constructed using QIAGEN Ingenuity Pathway Analysis https://www.qiagenbioinformatics.com/products/ingenuity-pathway-analysis/ accessed on 17 October 2022). Dashed lines represent an indirect relationship between molecules.

BMD treatment decreased the abundance of multiple proteins (FN1, FGA, FGG, and FGB), which are components of fibrinogen, and they influence the activation of monocytes and help in enhancing the host cellular defense [19]. Thus, they facilitate innate and T cell-mediated immune responses [20]. Acute-phase response proteins [complement C3 (C3), prothrombin (F2), alpha-1-acid glycoprotein (ORM1), inter-alpha-trypsin inhibitor heavy chain H4 (ITIH4), alpha-2-HS-glycoprotein (AHSG), complement C9 (C9), and complement regulatory protein variant 4 (CD46)] also decreased in abundance. The inflammatory cytokines IL-6, IL-1, IL-8 and TNF-alpha activate acute-phase response proteins in response to stress as part of a systemic acute-phase response contributing to local defense responses [21]. Likewise, overall the BMD effect leads to a decrease in the abundance of proteins such as TM9SF2 and MIA3, as well as ITIH3, involved in the regulation of actin cytoskeleton signaling induction of T cell activation, regulation of leucocyte migration, proinflammatory process facilitation respectively [22,23]. IPA summary for the overall BMD effect also showed IL4, IL6, and AGT inhibition (Figure 5). AGT or angiotensinogen regulates blood pressure, increasing it when the active form of AGT binds to its receptors. When proinflammatory cytokines are activated, hypertension is induced and related to increased levels of AGT [24]. Likewise, IPA canonical pathway demonstrates that in the inhibited GP6 signaling pathway, SYK, which influences innate immune response to bacteria pathogens, was downregulated. These results suggest that BMD acts in a manner that enables a decrease in the abundance of proteins that facilitates cytokines recruitment and associated inflammatory activities, thereby mitigating the cytokine storm that affects cell viability and functioning [25]. The IPA canonical pathway of proteins altered by BMD predicts that the ARE-mediated mRNA degradation pathway was activated. Among the proteins modified was the apoptosis-promoting protein TIA1, which was inhibited; however, AKT1, which deactivates components of the apoptotic machinery, was activated. Interestingly, the increase in the abundance of proteins involved in proteasome-mediated ubiquitin-dependent protein catabolic process provides evidence of a selective unfolded protein removal via a ubiquitin-mediated recognition system rather than through the autophagy-lysosome degradation pathway. Proteasome-mediated ubiquitin-dependent protein process is initiated during protein turnover, an action facilitated by multi-ubiquitin chain receptors (RAD23A and RAD23B) involved in the modulation of proteasomal degradation. With the use of inherent enzymes that connect chains of the polypeptide co-factor, ubiquitin binds to proteins to mark them for degradation [26]. This action is not only a quality control process of selectively eliminating abnormally folded proteins but also provides a source of amino acids that are useful in hypoxia conditions requiring gluconeogenesis, new protein synthesis, or ATP production [27]. Intriguingly, autophagy has been associated with the regulation of the transcription and secretion of proinflammatory cytokines such as IL-1α, IL-1β, and IL-18. Overall, the inhibition of the autophagy-lysosome degradation pathway provides evidence that BMD interrupts cytokine synthesis.

In our previous study, during *Salmonella* infection, lysosomal proteins and proteins involved in responding to cellular stress and repair of misfolded proteins increased in abundance [6]. In this study, the interaction between BMD treatment and the *Salmonella* challenge (BMD and SE interaction) decreased the abundance of proteins involved in protein folding, carbon metabolism, biosynthesis of nucleotide sugars, response to oxidative stress, positive regulation of NIK/NF-kappa signaling, and inflammatory response. The decrease in the abundance of CCT2 and CALR, as well as PPIA involved in folding newly synthesized proteins or misfolded proteins [28,29], may be attributed to the decrease in the number of unfolded proteins. The concentration of chaperones was increased in response to diverse stress and disease factors, which include unfolded proteins [30]. Moreover, the analysis of the top five IPA networks for interaction between BMD treatment and *Salmonella* challenges provides evidence of the mitigation of stress and an efficient protein production process. EFTUD2, which is predicted to have been activated, ensures cell cycle progression and prevents misfolding of proteins [31]. Likewise, LDHA aids in maintaining cell homeostasis by maintaining glycolysis, hence preventing cytotoxicity [32].

Additionally, BMD facilitates the increase in abundance of RNA binding proteins, spliceosome, peptidyl-prolyl cis-trans isomerase activity, protein transport, and cell adhesion. Notably, RBM23 is involved in pre-mRNA splicing and transcription, which facilitates the cell differentiation processes [33]. RPS27 is a ribosomal protein that interacts with DNA to initiate protein translation processes [34]. Also, CRNKL1 is involved in cell cycle progression as well as mRNA splicing [35]. There is also an increase in the abundance of laminins (LAMA5 and LAMC1), which are found in basement membranes and form the most abundant glycoproteins of the extracellular matrices of organisms [36]. More so, the increase in abundance of NID1, a component of the basement membrane shows efficient cell to cell interactions with the extracellular matrix. IPA upstream regulator analysis also predicted the inhibition of lipopolysaccharide, and it is reported that the lysis of bacteria cell wall by the host innate and adaptive immune response lead to the release of lipopolysaccharides, which triggers the activation of NF-κB and the production of inflammatory cytokines downstream [37,38]. In addition, there is an increase in the abundance of proteins involved in protein transport, hereby maintaining the cell homeostasis. Notably, VPS45 and FKBP3 of the immunophilin protein family provides a receptor for immunosuppressant such as rapamycin, an antibiotic preventing immune reaction that may trigger a response [39].

**Figure 6:**
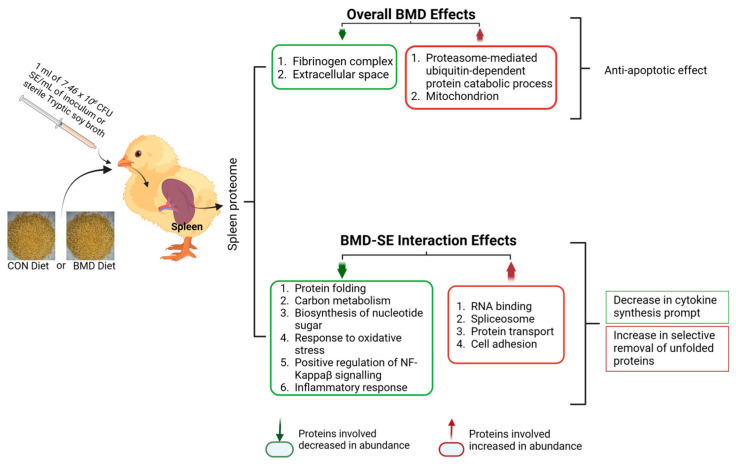
Summary of results of the effects of BMD treatments on the spleen proteome in chicks. The summary image was generated using BioRender (Toronto, ON, Canada) www.biorender.com, accessed on 2 April 2024.

In conclusion, BMD acts in a manner that inhibits processes that are associated with inducing inflammatory responses or cytokine recruitment such as autophagy and bacteria cell wall rupture. However, it ensures efficient mRNA splicing as well as protein synthesis processes that promote cell cycle progression and protein homeostasis in the presence of infection. While EFTUD2, and LDHA helps in maintaining cell and protein homeostasis, other key proteins that mediate BMD action include AKT1, which deactivates components of the apoptotic machinery, allowing RAD23A and RAD23B to facilitate a selective proteasome-mediated ubiquitin-dependent protein process for removal of damaged proteins. A summary of the spleen proteome in broiler chicks as influenced by BMD and BMD-SE interaction in shown in Figure 6.

## 4. Materials and Methods

### 4.1. Ethics Statement

The experimental protocol was approved by the Institutional Animal Care and Use Committee (IACUC; protocol no. 16-008) of North Carolina A&T State University before commencing animal studies.

### 4.2. Experiment Design and Salmonella Strain Used for the Experiment

One-day-old (n = 140) Ross 708 male broiler chicks were obtained from a commercial hatchery (Mountaire Farms, 1100 East 3rd Street, Siler City, NC 2734.), where they were vaccinated against Marek’s disease, infectious bursal, infectious bronchitis virus, and New Castle disease. Upon arrival, 20 chicks were randomly euthanized using CO_2_ asphyxiation, and ceca were aseptically removed and subjected to a five-day characteristic protocol for the detection of *Salmonella* spp. to confirm that the chicks were free of the nalidixic acid-resistant S. Enteritidis str. G1 (SE; marker strain) [6]. To perform this, ceca homogenates were sequentially cultured in sterile tetrathionate (TT) and Rappaport-Vassiliadis broths (RV; Remel Inc., Lenexa, KS), and xylose lysine Tergitol 4 (XLT4; Becton, Dickinson and Company, Sparks, MD) agar plates containing 50 μg/mL of nalidixic acid, as previously described [1]. Then, presumed *Salmonella* spp. colonies were isolated and biochemical confirmation was performed using transference into triple sugar iron (TSI; Remel Inc., Lenexa, KS) and lysine iron agar (LIA; Remel Inc., Lenexa, KS). The samples that were biochemically confirmed to be *Salmonella* were subjected to a serological latex agglutination test using polyvalent O antiserum reactive with serogroups A through I + Vi [40].

The remaining birds were randomly allocated into four groups: control with and without SE challenge (CON, n = 60; CON-SE, n = 60), Bacitracin Methylene Disalicylate with and without SE challenge (BMD, n = 60, BMD-SE, n = 60). Each treatment consisted of four replicate pens (n = 15 birds/ pen). The room temperature was set at 92 °F from day 1 to day 7, and at 87 °F from 8 to 14 days. Chicks were exposed to continuous lighting at 30 lux from placement to completion of study and given access to water and feed ad libitum. Feed consisted of an unmedicated (CON treatment) and a medicated corn–soybean meal basal starter diet (BMD inclusion rate of 0.055 g/kg) from 1 to 14 days (Table 6). Birds in the CON-SE and BMD-SE treatments were administered 1 mL of 7.46 × 10^8^ colony-forming units (CFU) SE /mL of inoculum (i.e., SE in Tryptic soy agar (TSA) broth) by oral gavage at one day of age. The CON and BMD treatments were gavaged with 1 mL of sterile TSA broth.

Diets used in this study included the following: (1) unmedicated corn-soybean meal (SBM) basal without BMD (Control diet); and (2) BMD diet in which Bacitracin Methylene Disalicylate was incorporated into unmedicated corn-SBM basal at 0.055 g/kg diet. Each of these diets were separately formulated for the starter (D 1 to 14) phases of broiler production cycle. Vitamin premix, supplied per kilogram of diet: vitamin A (6600 IU), vitamin D (1980 IU), vitamin E (33 IU), vitamin B12 (0.02 mg), biotin (0.13 mg), menadione (1.98 mg), thiamine (1.98 mg), riboflavin (6.60 mg), d-pantothenic acid (11.0 mg), vitamin B6 (3.96 mg), niacin (55.0 mg), folic acid (1.1 mg). Mineral premix, supplied per kilogram of diet: Manganese (Mn), 60 mg; Zinc (Zn), 60 mg; Iron (Fe), 40 mg; Copper (Cu), 5 mg; Iodine (I), 1.2 mg; Cobalt (Co), 0.5 mg.

### 4.3. Growth Performance Evaluation and Spleen Sample Collection

On day 3 or day 7 post-gavage, the spleen was collected aseptically from birds selected randomly from each treatment group (CON, n = 4/day; CON-SE, n = 4/day; BMD, n = 4/day; BMD-SE, n = 4/day) following euthanization by CO_2_ asphyxiation and stored at −80 °C.

### 4.4. Protein Extraction and Proteomic Analysis

Protein extraction and LC-MS/MS analysis were performed at Purdue University’s Proteomics Facility (Bindley Bioscience Center) in West Lafayette, Indiana. Spleen samples were homogenized in a 100 mM HEPES-KOH buffer using a bead beater. After transfer to a new tube, protein content was determined using a bicinchoninic assay. A total of 50 µg of protein was extracted from each sample and dissolved in a solution containing urea and dithiothreitol. The mixture was incubated at 37 °C for 1 h to break down disulfide linkages and alkylation of cysteine. After alkylation, the samples were dried and mixed with a solution containing ammonium bicarbonate and Pierce trypsin protease MS grade. The mixture was then digested overnight at 37 °C, after which the peptide digests were desalted using MicroSpin columns containing C18 silica and dehydrated using a hot vacuum centrifuge. The peptides were then stored at −80 °C pending further analysis [6].

#### 4.4.1. LC-MS/MS Analysis for Peptide Sequencing

Before the LC-MS/MS analysis, peptides were prepped by dissolving them in a mix of 3% acetonitrile and 0.1% formic acid, reaching a final concentration of 1 µg/µL. A total of 1 µL of this solution was used for the analysis. A well-established method [41,42] guided the peptide analysis using a sophisticated instrument setup. This setup combined a Dionex UltiMate 3000 RSLC nanosystem (ThermoFisher Scientific, Odense, Denmark) with a Q Exactive HF Hybrid Quadrupole-Orbitrap MS (Thermo Fisher Scientific, Waltham, MA, USA). The separation of the peptides happened at 40 °C and a flow rate of 200 nanoliters per minute. Two columns were used for this purpose: a trap column and an analytical column. Two mobile phases (liquids used for separation) were employed: A (water with 0.1% formic acid) and B (0.1% formic acid in 80% acetonitrile). A third solution (loading buffer) containing 2% acetonitrile and 0.1% formic acid was used to introduce the peptides. The peptides were loaded onto the trap column using the loading buffer for 5 min. Then, a gradually increasing concentration of mobile phase B (from 8% to 27% over 75 min) separated the peptides in the analytical column. The mobile phase B concentration was further increased to 45% at 100 min and then to 100% at 105 min for cleaning purposes. This high B concentration was maintained for 7 min before returning to the starting condition (2% B). The column was then equilibrated at 2% B for 130 min to prepare for the next sample. In between samples, the column went through a cleaning process involving three 30 min cycles with the LC gradient. The mass spectrometer functioned in a positive ion mode and a specific data acquisition mode (Top20). This mode selected the 20 most abundant ions for further analysis. A technique called higher-energy collision dissociation (HCD) was used to fragment the selected ions. The mass spectrometer had high resolution (120,000 for full MS1 and 15,000 for MS2 analysis). The analysis time was limited to 100 milliseconds for full MS1 and 20 milliseconds for MS2. Specific settings were used to control re-analysis of fragmented ions and the range of charges considered for the peptides. Additionally, the mass tolerance was set at a very high accuracy level (10 parts per million). The MS1 scanned a specific range (350–1600 *m*/*z*) to detect the peptides. The target automatic gain control (AGC) for MS1 was 3 × 10^6^, while for MS2 it was 1 × 10^5^ [6].

#### 4.4.2. Protein Identification and Quantification

The raw mass spectrometry data was imported into the MaxQuant 1.6.3.3 software. The resulting spectra were compared against the January 2020 Uniprot chicken (*Gallus gallus*) reference proteome database containing 2180 entries. Protein identification parameters included 10 ppm precursor mass tolerance, up to 2 missed cleavages for trypsin/Lys-C, methionine oxidation as a variable modification, and iodoethanol as a fixed modification. Proteins with LFQ values greater than 0 and a minimum of 2 MS/MS spectra were selected. Quantification of proteins was performed using the ‘unique plus razor peptides’ approach, where razor peptides are shared between protein isoforms.

For differential abundance analysis between control (CON) vs SE-challenged (CON-SE), and BMD-treated (BMD) vs BMD + SE (BMD-SE) groups, LFQ intensities were log_2_ transformed and missing values imputed. Analysis of variance (ANOVA) with false discovery rate (FDR) correction identified proteins altered by treatment or challenge. Contaminants, reverse sequences, and proteins not detected in ≥3 samples per group were removed. The Database for Annotation, Visualization, and Integrated Discovery (DAVID v6.8.) and The Ingenuity Pathways Analysis (IPA developed by QIAGEN Inc.) tools were used to identify the biological functions, canonical pathways, upstream analysis, and functional networks for differentially abundant spleen proteins [43] between BMD, BMD + SE, and appropriate controls [6].

## Figures and Tables

**Table 1:** Effect of *Salmonella* challenge on the concentration of SE in the ceca of broiler chickens.

Log_10_ CFU/g Cecal Contents		
Treatment	Day 3	Day 7
CON	ND	ND
CONSE	5.68 ± 0.36 ^a^	4.17 ± 0.18 ^a^
BMD	ND	ND
BMD-SE	3.39 ± 0.14 ^b^	2.41 ± 0.12 ^b^
*p*-value	<0.0001	<0.0001

^a, b^ Mean values bearing different superscript letters within a column are significantly different (*p* < 0.05).

**Table 5:** Top 5 Canonical pathways enriched with genes that increased and decreased in abundance in the spleen of broiler chickens fed with BMD with and without the presence of SE.

Ingenuity Canonical Pathways	−log (*p*-Value)	Ratio	Gene Symbols
OVERALL BMD EFFECT			
FAT10 signaling pathway	6.69	0.125	PSMA7, PSMB2, PSMB4, PSMC3, PSMC5, PSMD12, UBA6
Inhibition of ARE-mediated mRNA degradation pathway	6.36	0.0617	AKT1, PSMA7, PSMB2, PSMB4, PSMC3, PSMC5, PSMD12, TIA1, YWHAH, YWHAZ
Acute phase response signaling	5.83	0.0541	AKT1, C3, FGA, FGB, FGG, FN1, ITIH3, NOLC1, ORM1, RRAS2
Role of tissue factor in cancer	5.55	0.069	AKT1, FGA, FGB, FGG, FGR, GNA13, PIK3R4, RRAS2
GP6 signaling pathway	5.26	0.063	AKT1, COL12A1, COL6A1, FGA, FGB, FGG, PIK3R4, SYK
CHALLENGE X TREATMENT			
Splicesosomal cycle	5.24	0.122	CDC5L, EFTUD2, HSPA8, MAGOH, PPIE, U2AF2
Colanic acid building blocks biosynthesis	5.18	0.286	GFUS, GMPPA, GMPPB, GPI
GDP-mannose biosynthesis	4.82	0.5	GMPPA, GMPPB, GPI
TCA cycle II (eukaryotic)	4.26	0.174	CS, FH, MDH2, SUCLA2
Gluthathione redox reactions	3.85	0.138	GPX1, GPX3, GSTA1, PRDX6

score = 2log(*p*-value); {ratio = number of genes enriching set/total number of proteins in set}.

**Table 6:** Starter diet composition for the experiment.

Composition of Starter Diets (D1 to 14)		
	Control Diet	BMD Diet
Corn (7.5% Crude protein)	51.46	51.45
Soybean meal (47.5% Crude Protein)	40.39	40.40
Poultry fat	3.64	3.65
Limestone	1.07	1.07
Mono-Dicalcium phosphate	2.03	2.03
Salt NaCl	0.40	0.40
Sodium bicarbonate	0.02	0.02
L-Lysine HCl 98%	0.13	0.13
DL-Methionine 99.0%	0.34	0.34
L-Threonine 98.5%	0.11	0.11
NCSU Poultry Vitamin Premix ^2^	0.05	0.05
NCSU Poultry Mineral Premix ^3^	0.20	0.20
**Bacitracin (Antibiotic, g/kg)**	-	0.055
Choline chloride 60%	0.10	0.10
Selenium Premix	0.05	0.05
**Analyzed nutrient composition**		
Metabolizable energy (Kcal/kg)	3117	3131
Crude Protein, %	24.63	24.56
Crude Fat, %	4.74	5.03
Crude Fiber, %	2.3	2.4
Ash, %	6.32	6.15
**Calculated nutrient composition**		
Total Sulfur Amino Acids, %	1.03	1.03
Lysine, %	1.42	1.42
Calcium, %	0.96	0.96
Available phosphorus, %	0.48	0.48

## Data Availability

Data will be made available on reasonable request.

## Supplementary Materials

The following supporting information can be downloaded at: https://www.mdpi.com/article/10.3390/antibiotics13050414/s1.
